# Investigation on the pore characteristics of coal specimens with bursting proneness

**DOI:** 10.1038/s41598-019-52917-9

**Published:** 2019-11-11

**Authors:** Yutao Li, Yaodong Jiang, Bo Zhang, Honghua Song, Wenbo Dong, Pengpeng Wang

**Affiliations:** 1 School of Mechanics & Civil Engineering, China University of Mining and Technology (Beijing), Beijing, 100083 China; 2 State Key Laboratory of Hydroscience and Engineering, Tsinghua University, Beijing, 100084 China; 3 School of Energy & Mining Engineering, China University of Mining and Technology (Beijing), Beijing, 100083 China

**Keywords:** Tectonics, Mineralogy

## Abstract

To achieve further insight into the pore characteristics, the coal specimens with different bursting proneness before and after uniaxial compression failure are tested and compared in this paper. The data of mercury intrusion test is corrected by that of low-temperature nitrogen adsorption and desorption test (LTNAD). The pore size distribution and pore volume of specimens are obtained. The pore compressibility coefficient is determined based on the fractal dimension of pore. Scanning electron microscope (SEM) and computed tomography (CT) are combined to evaluated the pore connectivity. The value of pore compressibility coefficient of specimens with high bursting proneness is larger than that of medium bursting proneness. It means more compressibility and abrupt failure under stress. The researches of both SEM and CT indicate that the pore connectivity of specimens with medium bursting proneness is better. The results show that great differences exist in the pore characteristics of specimens with high and medium bursting proneness, and uniaxial compression failure exacerbates the complexity of pore characteristics.

## Introduction

In China coal occupies a dominant position in energy, burst is one of the most frequent and destructive forms of dynamic disasters in coal mines. It is the sudden release of elastic strain energy of coal, which causes great damage to engineering structures and mechanical equipment, has been a serious threat to human safety^1–3^. The mechanism of burst is complex and there are many influencing factors, including the intrinsic physical and mechanical properties of coal, external engineering geological conditions and site construction conditions, such as the thickness of overburden strata greater than 500 feet (151.5 meters), a strong floor rock that does not heave readily, mountainous terrain, steeply dipping beds, and similar geologic structures where localized stress concentration exists, stress concentration caused by unreasonable mining methods^4–6^.

Coal is plant remains subjected to a prolonged and complicated process of biological, chemical, and tectonic factors under high temperatures and pressures, which is an anisotropic rock with a sophisticated pore structure^7–9^. Under different stress and strain environments, the interaction of primary pore and secondary pore induced by inelastic deformation produces great changes in porosity, permeability, adsorption, and desorption properties of coal^10^. The changes of pore characteristics have an important influence on the properties of materials. Chen *et al*.^11^ considered that the properties of porous materials are closely related to the characteristics of pore structure. Under quasi-static or dynamic loading condition, compressive strength of concrete decreases with the increasing porosity. Lee *et al*.^12^ considered that the compressive strength of porous materials decreased with the increase of porosity to highly porous mullite ceramics. Liu *et al*.^13^ considered that coal deformation and gas migration are closely related to pore characteristics.

Experts and scholars from domestic and abroad have made an effort to characterize pore structure and attained valuable results. Jiang *et al*.^14^ and Yu *et al*.^15^ analyzed the method of age and maximum entropy estimation. Okolo *et al*.^16^ elaborated the pore characteristics of coal including the surface area and porosity by small angle X-ray scattering and mercury intrusion porosimetry. Wang *et al*.^17^ used fractal dimension and image analysis technology to characterize the pore and fracture characteristics in low-rank coal samples and the influence of pore structure parameters on gas permeability of coal samples. Bhatia^18^ revised the random-pore model, the solid consisted of spherical microporous grains and the micropores in each grain. The model gave the most adequate correlation with experimental data for various coals. Zhu *et al*.^19^ analyzed the pore surface area, pore volume and pore compressibility of gas outburst coal and bursting proneness coal by mercury intrusion test and low-temperature nitrogen adsorption and desorption test (LTNAD), and concluded that there were significant differences in pore structure characteristics between them. Li *et al*.^20^ characterized the heterogeneity of low porosity and low permeability in naturally fractured rock specimens by mercury intrusion experiment and fractal dimension calculation. Zhang *et al*.^21^ developed the steady-state swelling model based on Gibbs adsorption model, approved that adsorption and desorption would occur between coal and adsorbate, and the main way for adsorbate to enter coal is pore system. Zhao *et al*.^22^ measured the pore size distribution of six coal specimens by nuclear magnetic resonance cryoporometry technology and LTNAD, and proved that both techniques could describe the pore characteristics of coal specimens. Cinefra^23^ and Nasihatgozar^24^ demonstrate the influence of nanoparticles on the visco-embedded nanoplate and concrete beams. Liu *et al*.^25^ investigated the relationship of pore structure and methane adsorption capacity based on scanning electron microscopy (SEM). Zhao *et al*.^26^ studied the pore structure characterization of coal through synchrotron radiation nano-CT and proved the anisotropy of permeability.

In this paper, the pore characteristics of specimens with high bursting proneness (Hongqinghe Coal Mine) and medium bursting proneness (Nalinhe Coal Mine) before and after uniaxial compression failure are described by mercury intrusion test and LTNAD. The data of mercury intrusion test is corrected by the data of LTNAD, and then used to quantitatively describe the pore size distribution and porosity. Pore compressibility coefficients are calculated. Scanning electron microscope (SEM) and computed tomography (CT) are combined to evaluated the pore connectivity.

## Experiments

### Specimens preparation

**Figure 1:**
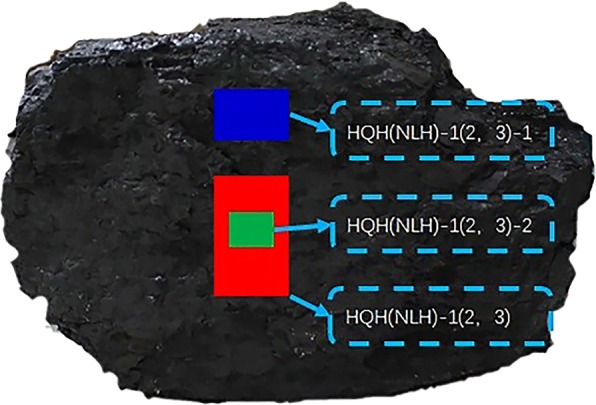
Schematic diagram of the sampling-region of coal specimen.

High bursting proneness specimens tested were collected from No. 9 coal seam in the Hongqinghe Coal Mine at the average buried depth of 760 m. Medium bursting proneness specimens tested were collected from No. 3 coal seam of Nalinhe Coal Mine at the average buried depth of 600 m. All the specimens were carefully transported to the laboratory, and stored under environment-controlled conditions for weathering protection until the initiation of experiment. The uniaxial compression test was carried out firstly. The specimens were standard cylinder with diameter of 50 mm and height of 100 mm, and numbered HQH-1(−2, −3) and NLH-1(−2, −3). The specimens were core-drilling, and the two ends of the specimens are ground flat on the flattening machine. It ensured that the surface of both sides of the specimens were parallel and smooth without large scratches, and the degree of non-parallelism between the two ends should not be greater than 0.01 mm, and the deviation between the diameters of the upper and lower ends should not be greater than 0.02 mm. The mercury intrusion test and LTNAD were carried out subsequently. Specimens in the two tests were different from the former. They were divided into two categories. The No.1 part was extracted from lump coal directly. The sampling-region was the same as the specimens of uniaxial compression test but right above of them, and numbered HQH-1-1, HQH-2-1, HQH-3-1 and NLH-1-1, NLH-2-1, NLH-3-1, respectively. The No. 2 part was extracted from the central region of fracture propagation zone after uniaxial compression failure, and numbered HQH-1-2, HQH-2-2, HQH-3-2, and NLH-1-2, NLH-2-2, NLH-3-2, respectively, as shown in Fig. 1. The specimens were crushed to particles of about 1–2 cm in diameter and 5 g in mass. Before the uniaxial compression test, the axial wave velocity test and industrial analysis of specimens were carried out, and the results were shown in Table 1.

**Table 1:** Results of industrial analysis and axial wave velocity test of coal specimens.

Sampling location	Fixed carbon (%)	Ash content (%)	Volatile matter (%)	Moisture (%)	axial wave velocity (m/s)
Hongqinghe	56.59	7.17	28.20	8.05	850–950
Nalinhe	37.26	18.11	38.57	6.05	850–920

### Monitoring plan

The uniaxial compression test was conducted on the GCTS comprehensive test system, and it had dynamic and static closed-loop digital electro-hydraulic servo control function. The loading process was controlled by displacement of 0.002 mm/s. The deformation was recorded by the circumferential strain gauge. After the maximum stress, the coal specimens would be quickly unloaded and protected by thermal contractible plastic sleeve. It would be profitable to observe the development of surface fractures after damaged and sample for the successive test.

The mercury intrusion test was performed on AutoPore IV 9500 Micromeritics Instrument. The maximum of working pressure was 60000 psi (414 MPa), and the pore size measuring range was 3 nm–1000 μm. Before the test, the specimens were dried for 12 hours at 373 K, and then evacuated from the low-pressure port to <50 μm Hg for 5 minutes. The purpose was to remove residual gas and moisture in the coal specimens. According to the suggestions of Gan *et al*.^27^ and Zhao *et al*.^28^, the contact angle between mercury and pore surface was set to 130 degrees, and the surface tension of mercury was set to 0.485 J/m^2^.

The LTNAD was completed on ASAP 2460 physical adsorption device, which was produced by Micromeritics Company. The pore size tested measuring range of the equipment was 1–300 nm. The residual gas and moisture in specimens were removed before the test, and the method was the same as mercury injection test. During the test, the temperature was 77 K, and the relative pressure (P/P_0_) was no more than 0.996. The pore size distribution and volume of coal specimens were determined by Barrett–Joyner–Halenda (BJH) method^29^.

Scanning electron microscope (SEM) and computed tomography (CT) were finished at State Key Laboratory Coal Resources and Safe Mining of China University of Mining & Technology, Beijing. The specimens were scanned by SEM-Servopulser scanner, magnification included 35, 50, 100, 200, 500, 750, 1000. The cylindrical specimens with diameter of 50 mm and height of 100 were scanned using an ACTIS300-320/225 scanner manufactured by BIR Corporation, USA. The scanning time is 15 s per slice with slice thickness and slice spacing both of 0.05 mm. The 3-D reconstructed model was built by Mimics. Mimics is a modular software and an image control system invented by Materialise.

## Test Results

### Stress-stain curves of coal specimens with bursting proneness

**Figure 2:**
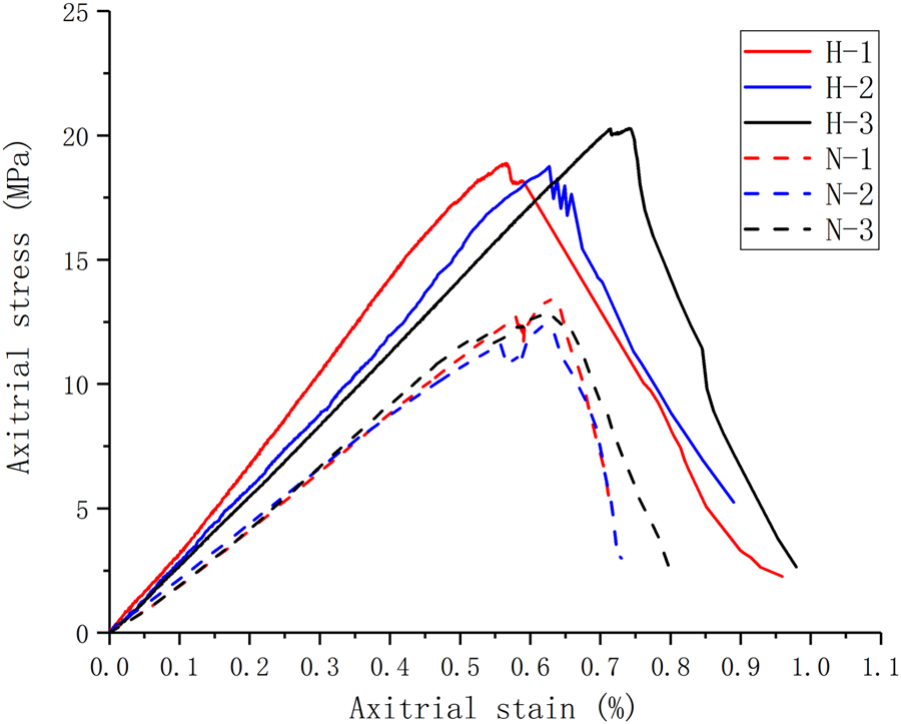
Stress-strain curves of the two groups of specimens.

In uniaxial compression test, the stress-strain curves of coal specimens were shown in Fig. 2. The stress-strain curves of two groups of specimens both included four stages: micro-porous fracture compaction stage, elastic deformation stage, secondary micro-porous fracture initiation and stable expansion stage, and post-peak micro-porous fracture rapid expansion stage. The stages were consistent with Xue’s research^30^. The average uniaxial compressive strength and elastic modulus of specimens from Hongqinghe Coal Mine were 19.293 MPa and 3.169 GPa, respectively. The average uniaxial compressive strength and elastic modulus of specimens from Nalinhe Coal Mine were 12.115 MPa and 2.251 GPa, respectively. It could be seen that great differences existed in the process of energy accumulation and dissipated between the two groups of specimens. Under the same stress, the specimens with high bursting proneness had larger deformation and stronger compressibility. After the maximum stress, the specimens approached to abrupt failure. After the first stress reduction of the specimens with medium bursting proneness, the stress continued to increase, the process of energy dissipation is more complex, and the specimens approached to gradual failure.

### The mercury intrusion test and LTNAD

The pore parameters of specimens based on the mercury intrusion test and LTNAD were shown in Table 2. The porosity of specimens from Nalinhe Coal Mine were higher than that of the Hongqinghe Coal Mine both before and after uniaxial compression failure, but the pore specific area and median pore diameter were lower than the former. The reason was that the quantity of different pore diameters from the two groups of specimens varied greatly. The bulk density and accumulative density were higher than the former. It indicated that the mineral composition of the two groups of specimens was quite different.

**Table 2:** Pore parameters of the two groups of specimens.

Specimens ID	Porosity (%)	Specific surface (m^2^/g)	Median pore diameter (nm)	Bulk density (g/ml)	Accumulative density (g/ml)
HQH-1-1	10.239	28.769	11.80	1.208	1.352
HQH-1-2	10.735	33.275	13.00	1.180	1.346
HQH-2-1	12.338	35.247	15.01	1.163	1.350
HQH-2-2	12.698	29.261	14.92	1.160	1.343
HQH-3-1	13.403	42.003	10.11	1.285	1.507
HQH-3-2	13.733	43.601	10.52	1.262	1.458
NLH-1-1	13.627	32.351	12.33	1.366	1.582
NLH-1-2	14.425	33.477	12.73	1.354	1.582
NLH-2-1	14.240	38.879	11.16	1.361	1.535
NLH-2-2	14.686	36.470	11.84	1.353	1.531
NLH-3-1	13.976	30.929	12.22	1.429	1.664
NLH-3-2	14.139	33.411	11.80	1.374	1.579

**Figure 3:**
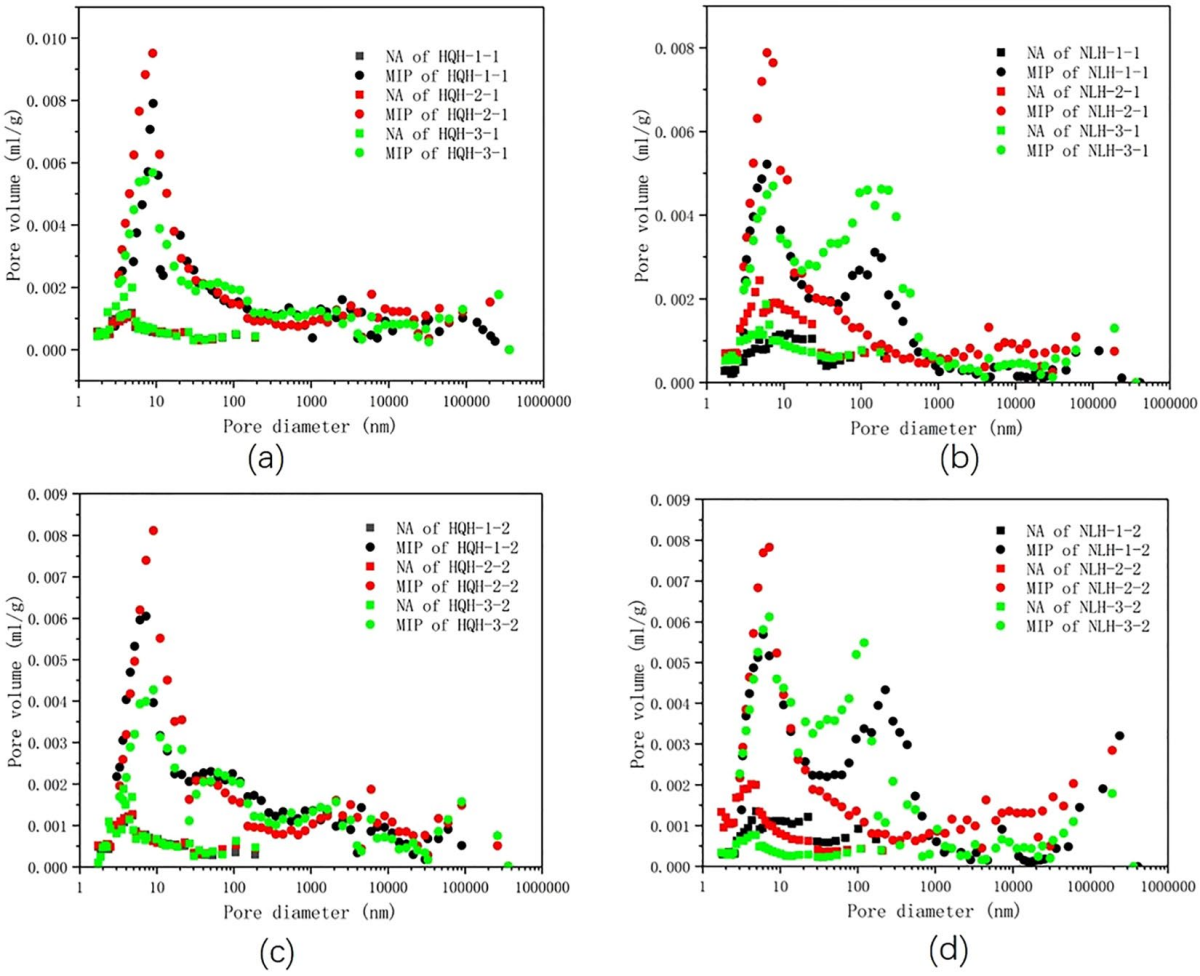
The pore size distribution of specimens from LTNAD and MIP data before and after uniaxial compression. (**a**) specimens from Hongqinghe Coal Mine before uniaxial compression failure; (**b**) specimens from Nalinhe Coal Mine before uniaxial compression failure; (**c**) specimens from Hongqinghe Coal Mine after uniaxial compression failure; (**d**) specimens from Nalinhe Coal Mine after uniaxial compression failure.

The pore size distribution of specimens before and after uniaxial compression failure were shown in Fig. 3. NA represented the result of LTNAD, and MIP represented the result of mercury intrusion test. The pore size measuring range of equipment were 1 to 300 nm (in LTNAD) and 30 nm to 1000μm (in mercury intrusion test), and the overlap was 30–300 nm. The result in mercury intrusion test was much larger than that of LTNAD. The main reason was that the high working pressure in mercury intrusion test brought the compression deformation of coal matrix and pore, and the increment of pore volume, these distorted the result. This was consistent with the previous study^31^. In LTNAD, relative pressure was low (P/P_0_ < 1, P refers to working pressure, P_0_ refers to the saturation vapor pressure), and partial pore could not adsorb fully. The result of pore volume was distorted either. It was reasonable to correct the result in mercury intrusion test based on the result in LTNAD.

## Comprehensive Description of Pore characteristics

### Pore volume

According to the previous studies, a combined pore classification was employed in this paper: microfracture (d > 10,000 nm), macropore (1000 < d < 10,000 nm), mesopore (100 < d < 1000 nm), transition pore(10 < d < 100 nm), micropore (2 < d < 10 nm) and super-micropore (d < 2 nm), where d was pore diameter^32,33^. Researches demonstrated that the working pressure in mercury intrusion test had an obvious influence on the results because of the pore compressibility, and the results should be corrected when the pressure was bigger than 10 MPa^34^.

Here would introduce the correcting method. Since the volume of micropore and transition pore were quite different between result of the two tests in Fig. 3, the result of mercury intrusion test was corrected based on the data of LTNAD, and the full-scale pore size distribution and pore compressibility in coal specimens were obtained ultimately.

In porous compressible media, the increment of pore volume measured by mercury intrusion test can be described as^35^:

1 $$\Delta {V}_{obs}=\Delta {V}_{p}+\Delta {V}_{c}$$

where *ΔV*_*obs*_ refers to the increment of pore volume. *ΔV*_*p*_ refers to the increment of pore-filling volume. The super- micropore and micropore in the specimens could not be filled in the mercury intrusion test, even under high pressure, while they could be obtained by the data of LTNAD. *ΔV*_*c*_ refers to the increment of pore volume caused by the compressibility of coal matrix.

**Figure 4:**
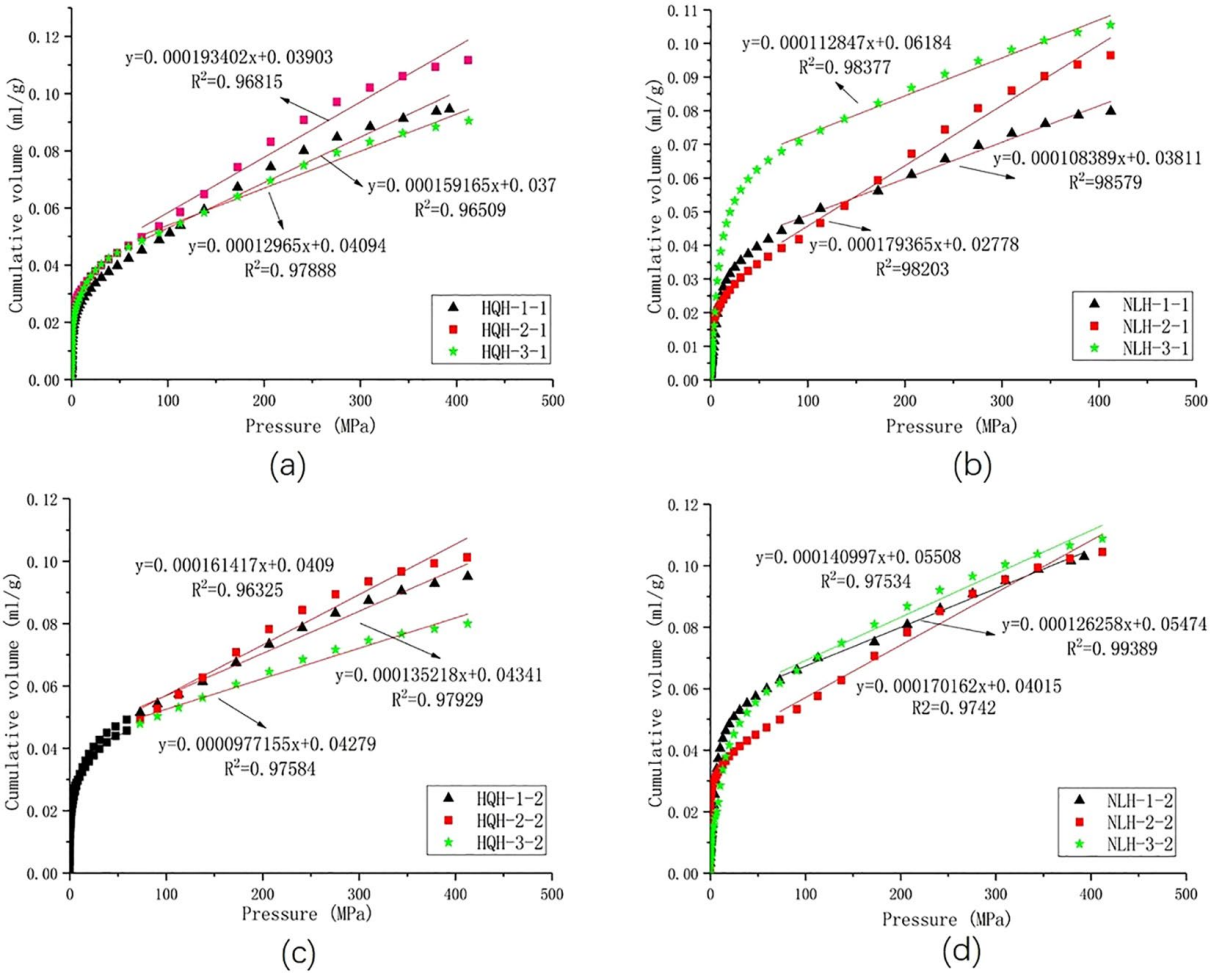
Plots of cumulative volume of every specimen as a function of pressure before and after the uniaxial compression failure and linear regression of pore volume versus pressure. (**a**) specimens from Hongqinghe Coal Mine before uniaxial compression failure; (**b**) specimens from Nalinhe Coal Mine before uniaxial compression failure; (**c**) specimens from Hongqinghe Coal Mine after uniaxial compression failure; (**d**) specimens from Nalinhe Coal Mine after uniaxial compression failure.

An excellent linear relationship could be obtained in the plots of the observed pore volume versus mercury intrusion pressure when the pressure is high, as shown in Fig. 4.

According to Eq. (1), $\frac{\Delta {V}_{C}}{\Delta P}$ could be further represented as

2 $$\frac{\varDelta {V}_{C}}{\varDelta P}\approx \beta -\frac{{\sum }_{3nm}^{17nm}\varDelta {V}_{p}}{\varDelta P}$$

where *β* refers to *ΔV*_*obs*_/*ΔP*, which was approximately considered as a constant under high pressure, and equaled to the slope of the fitting line in Fig. 4. *ΔP* refers to the increment of mercury intrusion pressure. $\mathop{\sum }\limits_{3nm}^{17nm}\varDelta {V}_{p}$ refers to the pore volumes of 3–17 nm in diameter, the corresponding pressure was larger than 60 MPa in this paper. The fitting results were the best in this interval both before and after the uniaxial compression failure for specimens, and the correlation coefficients were all 0.98 or so, as shown in Fig. 4. $\,\mathop{\sum }\limits_{3nm}^{17nm}\varDelta {V}_{p}$ were obtained from the data of LTNAD.

**Figure 5:**
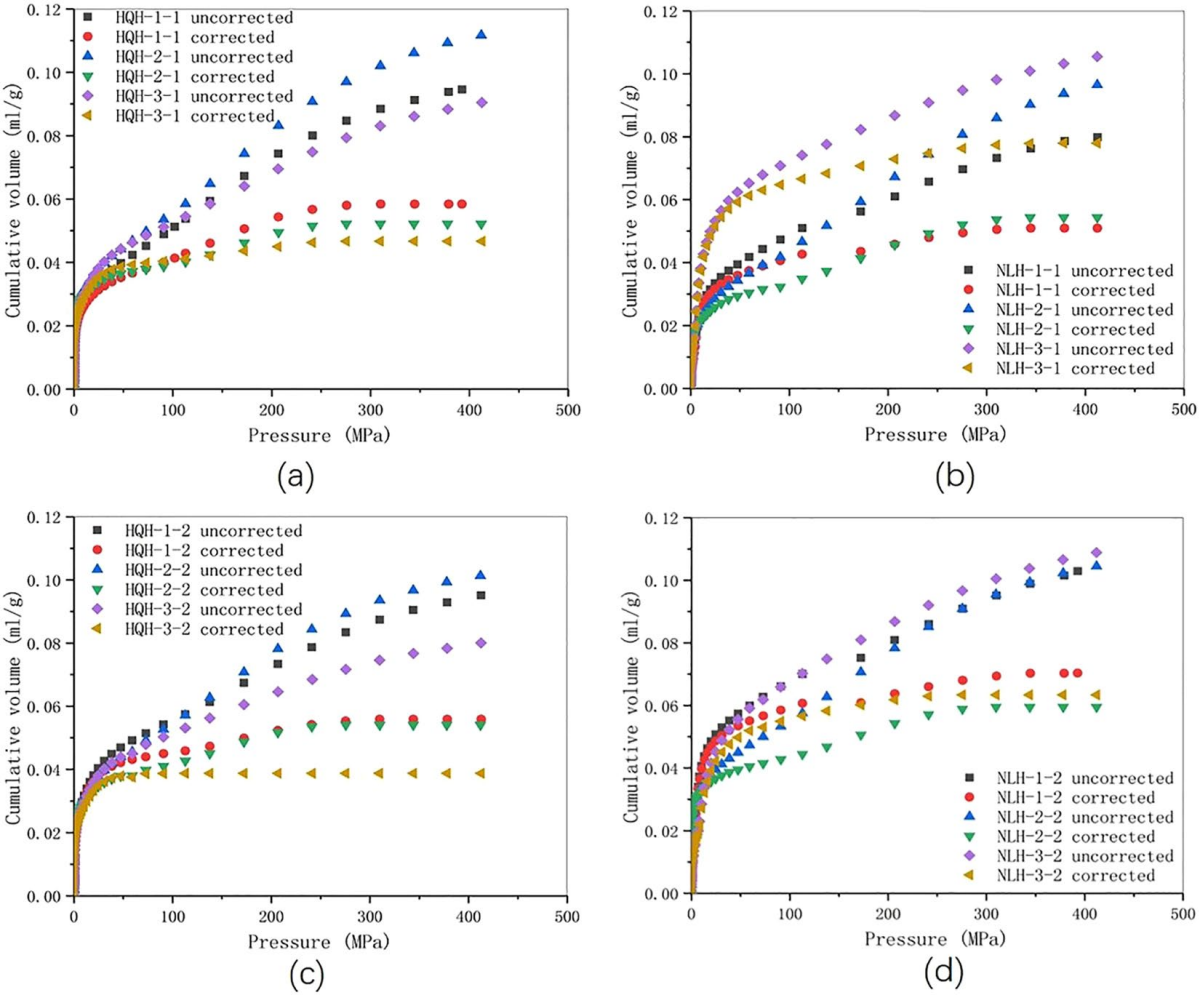
Comparison of observed mercury intrusion volume before and after correction. (**a**) specimens from Hongqinghe Coal Mine before uniaxial compression failure; (**b**) specimens from Nalinhe Coal Mine before uniaxial compression failure; (**c**) specimens from Hongqinghe Coal Mine after uniaxial compression failure; (**d**) specimens from Nalinhe Coal Mine after uniaxial compression failure.

Both the original accumulated pore volume and the corrected accumulated pore volume calculated by Eq. (2) were presented in Fig. 5. The correction value of specimens from Hongqinghe Coal Mine before uniaxial compression failure was larger than that of Nalinhe Coal Mine. The curve after correction was more upward, indicating that the compression deformation was larger. After uniaxial compression failure, the pore volume of two groups of specimens increased slightly, and HQH-3-2 specimens data appeared discrete. The remaining data showed that specimens from Nalinhe Coal Mine had larger pore volume. It indicated that specimens from Hongqinghe Coal Mine had more deformation under high pressure, which was coincided with result the of uniaxial compression test.

**Figure 6:**
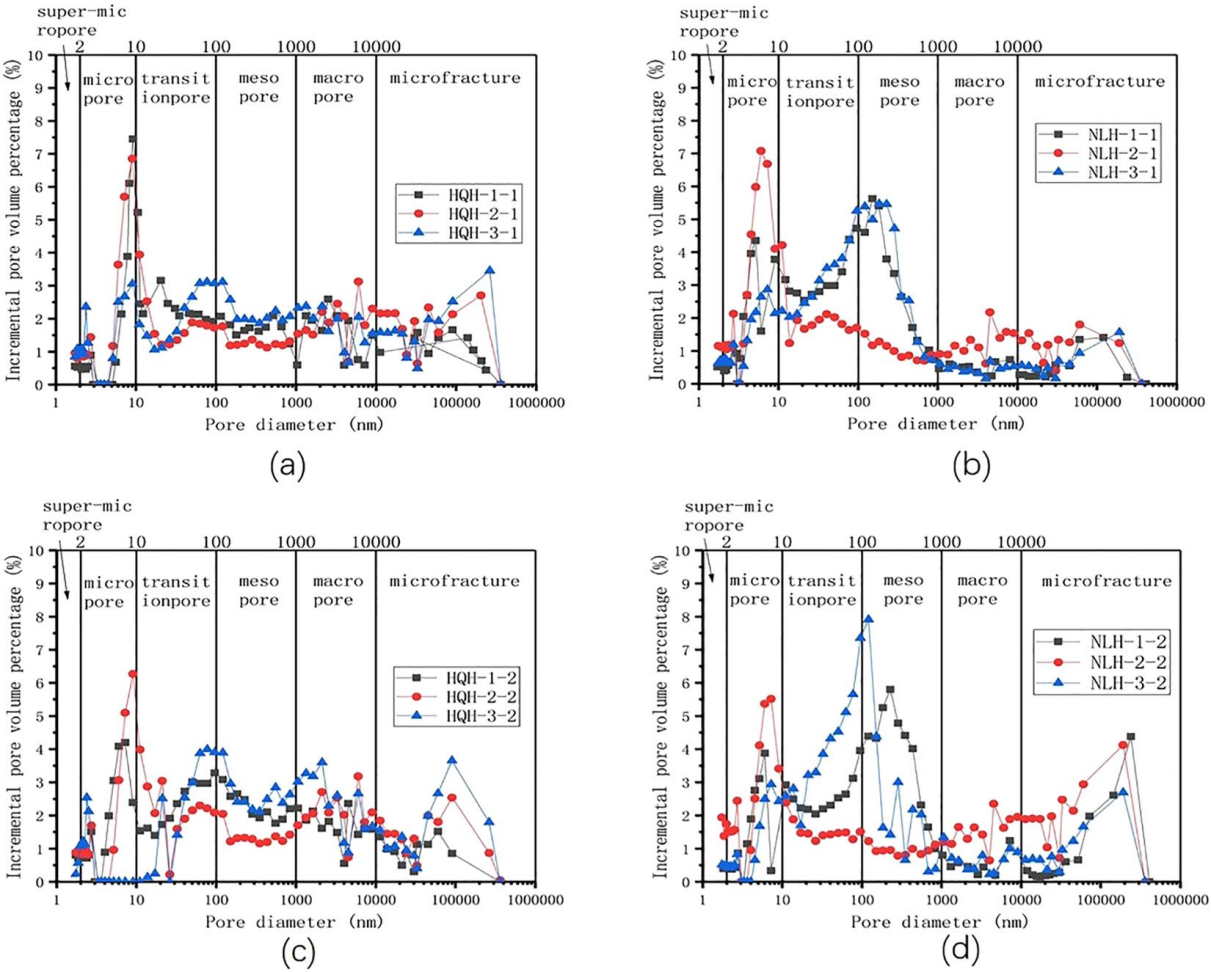
Plots of pore size distribution of the two groups of specimens before and after the uniaxial compression failure. (**a**) specimens from Hongqinghe Coal Mine before the uniaxial compression failure; (**b**) specimens from Hongqinghe Coal Mine after the uniaxial compression failure; (**c**) specimens from Nalinhe Coal Mine before the uniaxial compression failure; (**d**) specimens from Nalinhe Coal Mine after the uniaxial compression failure.

Before and after uniaxial compression failure, the pore size distribution of two groups of specimens was shown in Fig. 6. It could conclude that the pore size distribution of specimens from Hongqinghe Coal Mine was more complicated. The quantity of transition pore (10 < d < 100 nm), mesopore (100 < d < 1000 nm) and macropore (1000 < d < 10,000 nm) were great. After the uniaxial compression failure, the total pore volume remained approximately (HQH-1 changes from 0.06118 to 0.06011 ml/g; HQH-2 changes from 0.05649 to 0.05867 ml/g; HQH-3 data is discrete), but the volume of micro fracture and micro-pore decreased, and the volume of macropore, mesopore and micro-pore increased. The increase or decrease in quantity of transition pore was uncertain because the uniaxial compression destroyed the original structure of coal matrix, expanded microcracks into cracks, and produced new super-micropore. The pore size distribution of specimens from Nalinhe Coal Mine mainly concentrated on micropore, transition pore and mesopore. The total pore volume changed greatly after uniaxial compression failure (NLH-1 changed from 0.05357 to 0.07305 ml/g, NLH-2 from 0.06045 to 0.06894 ml/g, NLH-3 from 0.08276 to 0.06616 ml/g). This indicated that the deformation of various parts in specimens was quite different. The volume of microfractures and macropore increases greatly, while the volume of micropore decreases.

### Mercury intrusion and extrusion curves

**Figure 7:**
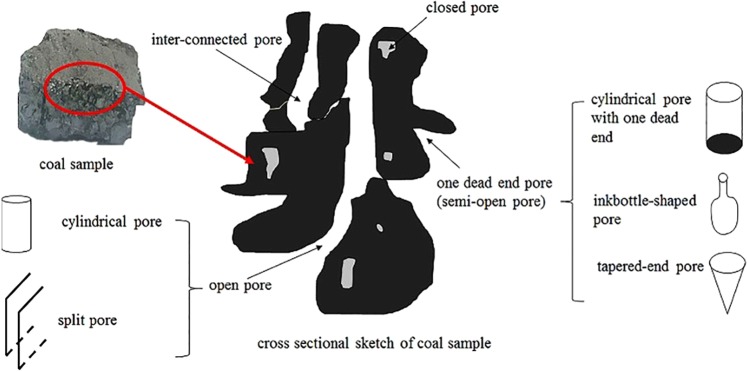
Sketch of the shape of pore in specimens^36^.

According to the previous study, the pore could be classified open pore and closed pore. Open pore included inter-connected pore, passing pore, and one dead end pore (namely semi-open pore). The passing pore could be divided into cylindrical pore and split pore. Semi-open pore could be divided into cylindrical pore with one dead end, inkbottle-shaped pore and tapered-end pore, as shown in Fig. 7 ^36^.

**Figure 8:**
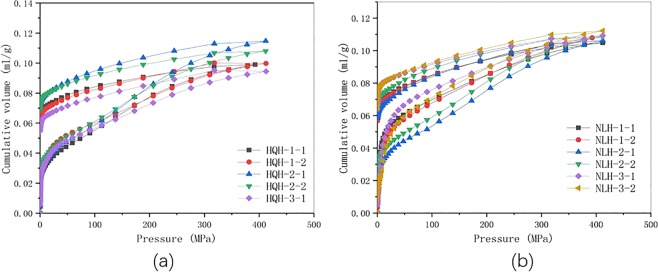
The mercury intrusion and extrusion curves of the two groups of specimens before and after uniaxial compression failure. (**a**) specimens from Hongqinghe Coal Mine; (**b**) specimens from Nalinhe Coal Mine.

The mercury intrusion and extrusion curves of specimens before and after uniaxial compression failure were shown in Fig. 8 (the HQH-3-2 data was discrete and omitted). Hysteretic loops exited between intrusion curve and extrusion curve. Generally, the existence of hysteretic loops was due to the existence of open pore in specimens, and the pore-throat structure of inkbottle-shaped pore leaded to the convex phenomenon in mercury extrusion curves (at 320 MPa of pressure in the test). The larger the width of hysteretic loops, the larger the difference of pore volume between mercury intrusion and mercury extrusion under the same pressure. It signified that the more open pore existed. To specimens from Hongqinghe Coal Mine, the width of hysteresis loops was larger than that of Nalinhe Coal Mine. The convexity phenomenon of mercury intrusion and mercury extrusion curves was more obvious than that of Nalinhe Coal Mine. These demonstrated that more inkbottle-shaped pore and capillary phenomena occurred. However, the increased or decreased width of the hysteretic loops of different specimens in the same group demonstrated the variability of pore size distributions. The uniaxial compression failure changed the width of hysteretic loops slightly, because the size of specimens in the mercury intrusion test was small (1–2 cm in diameter). The existing research could only statistically prove that the mercury intrusion and extrusion curves of specimens with different bursting proneness were different, and the volume of pore with different size varied irregularly before and after uniaxial compression failure.

### Pore compressibility

Under high mercury intrusion pressure, the elastic-plastic deformation of coal matrix occurred, and the pore changed inevitably. These were displayed in the non-linear increase of cumulative mercury intrusion volume during the mercury intrusion stage and the different width of hysteresis loop. Because of the significant impact on the result of mercury intrusion test, the pore compressibility of two groups of specimens with different bursting proneness was further analyzed in this paper.

According to Menger Model and mercury intrusion test^37^, computational formula of the pore compressibility coefficient of coal specimens was shown as Eqs (3) and (4):

3 $${k}_{p}=\frac{1}{{V}_{P}}\ast \frac{d{V}_{P}}{dP}$$

4 $$\frac{d{V}_{P}}{dr}=-\frac{P}{r}\ast \frac{d{V}_{P}}{dP}$$

where *V*_*P*_ refers to the mercury intrusion volume, approximately equals to the sum volume of pores with diameter of greater than specified value (2r, r equals the pore radius). $\frac{d{V}_{P}}{dP}$ refers to the functional relation between the mercury intrusion volume and working pressure.

The relationship between mercury intrusion pressure and pore diameter satisfies Washburn Equation:

5 $${\rm{P}}=-\,2{\rm{\sigma }}\ast \,\cos \,{\rm{\theta }}/{\rm{r}}$$

where P refers to the mercury intrusion pressure. σ refers to the mercury surface tension (0.485 J/m^2^ in this test). θ refers to the contact angle of mercury(130°in this test), r refers to the pore radius.

The relationship between pore volume, pore radius and fractal dimension of pore volume was shown in Eq. (6):

6 $$\frac{d{V}_{P}}{dr}\propto -{r}^{2-D}$$

where D refers to fractal dimension.

Equation (7) was derived from Eqs (4–6):

7 $$\frac{d{V}_{P}}{dP}\propto {P}^{D-4}$$

Finally, calculating the logarithm of both sides of Eq. (7) could obtain the Menger Model, as shown in Eq. (8):

8 $${\rm{lg}}\,\frac{d{V}_{P}}{dP}\propto (D-4)lgP$$

**Figure 9:**
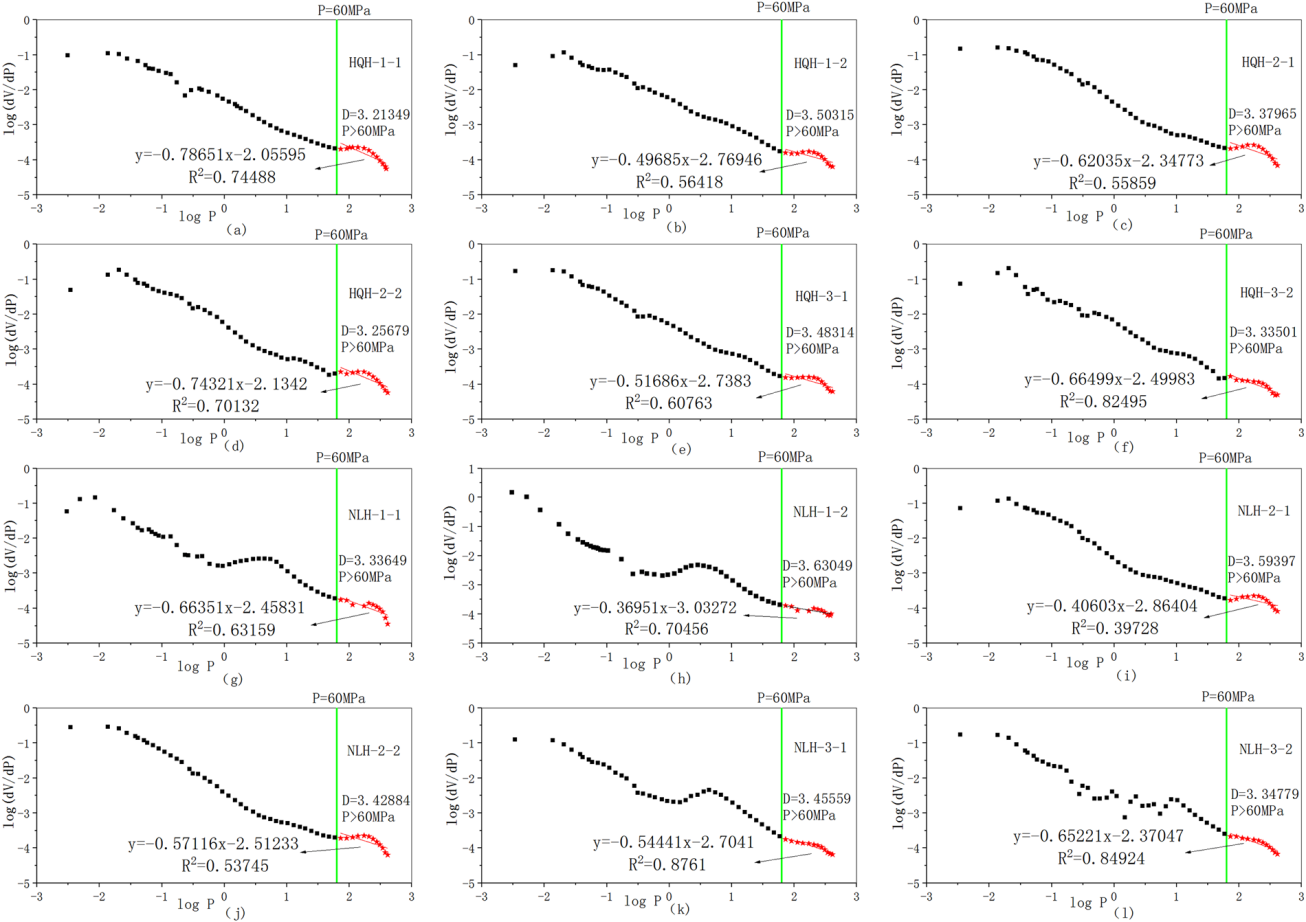
Plots of ${\rm{lg}}\,\frac{d{V}_{P}}{dP}$and lgP of the two groups of specimens before and after uniaxial compression failure. (**a**,**c**,**e**) specimens from Hongqinghe Coal Mine before uniaxial compression failure; (**b**,**d**,**f**) specimens from Hongqinghe Coal Mine after uniaxial compression failure; (**g**,**i**,**k**) specimens from Nalinhe Coal Mine before uniaxial compression failure; (**h**,**j**,**l**) specimens from Nalinhe Coal Mine after uniaxial compression failure.

The fractal dimension values(D) of different pores were calculated by the slopes of fitting lines between ${\rm{lg}}\,\frac{d{V}_{P}}{dP}$ and lg P. The high-pressure stage was selected to research, the relationship between ${\rm{lg}}\,\frac{d{V}_{P}}{dP}$and lgP was shown in Fig. 9.

Equation (9) was derived from Eq. (8):

9 $${V}_{P}=a+b{P}^{D-3}$$

Equation (10) was derived from Eqs (3) and (9):

10 $${k}_{P}=\frac{b(D-3){P}^{D-4}}{a+b{P}^{D-3}}$$

where constant of a and b were the intercept and slope of the fitting line in Fig. 4, respectively.

**Figure 10:**
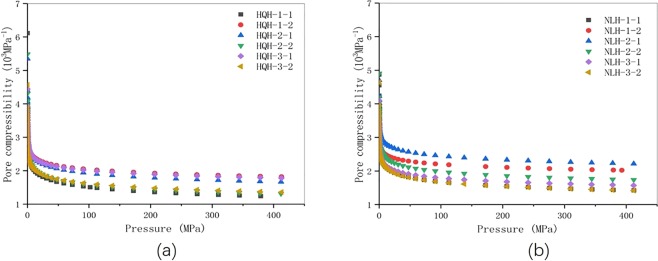
Plots of the pore compressibility coefficient and pressure of the two groups of specimens before and after uniaxial compression failure. (**a**) specimens from Hongqinghe Coal Mine; (**b**) specimens from Nalinhe Coal Mine.

The pore compressibility coefficient was calculated and showed in Fig. 10. Before and after uniaxial compression failure, the pore compressibility coefficient of specimens from Hongqinghe Coal Mine was bigger than that of Nalinhe Coal Mine. It indicated that the pore deformation was bigger and the pore compressive property was better to the specimens from Hongqinghe Coal Mine under the same stress. Before uniaxial compression failure, the pore compressibility coefficient of specimens from Hongqinghe Coal Mine changed from 86.69 to 0.01 × 10^−3^ MPa^−1^, and that of Nalinhe Coal Mine changed from 44.86 to 0.01 × 10^−3^ MPa^−1^. The mercury intrusion pressure changed from 0.003 to 410 MPa correspondingly. After uniaxial compression failure, the pore compressibility coefficient of specimens from Hongqinghe Coal Mine changed from 68.76 to 0.01 × 10^−3^ MPa^−1^, and that of Nalinhe Coal Mine changed from 46.31 to 0.01 × 10^−3^ MPa^−1^. The results showed that pore volume of specimens from Hongqinghe Coal Mine was less, the distribution was more sophisticated and the quantity of open pore were bigger.

### Pore connectivity

**Figure 11:**
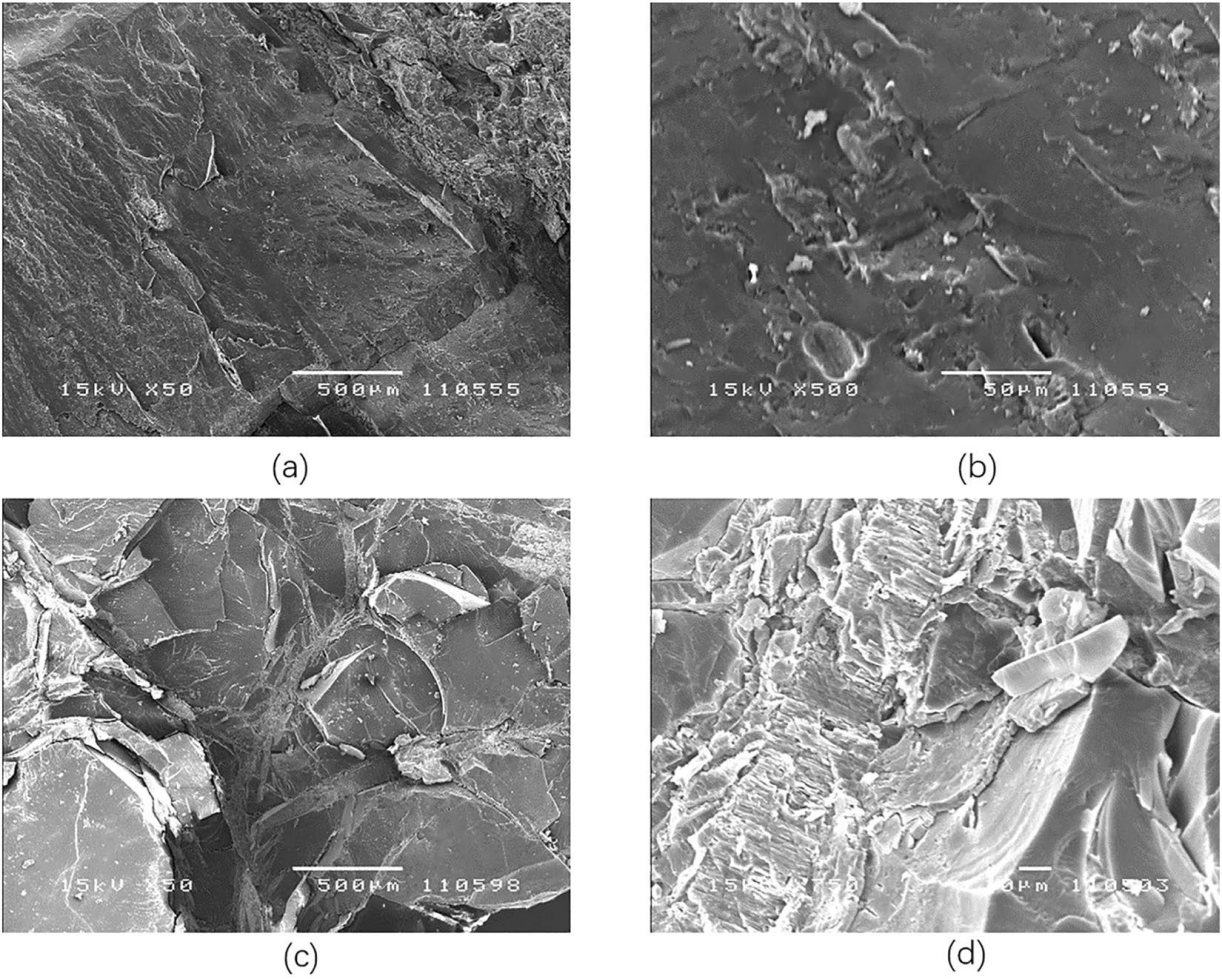
Images obtained by SEM. (**a**) image of Hongqinghe Coal Mine with magnification of 50; (**b**) image of Hongqinghe Coal Mine with magnification of 500; (**c**) image of Nalinhe Coal Mine with magnification of 50; (**d**) image of Nalinhe Coal Mine with magnification of 750.

**Figure 12:**
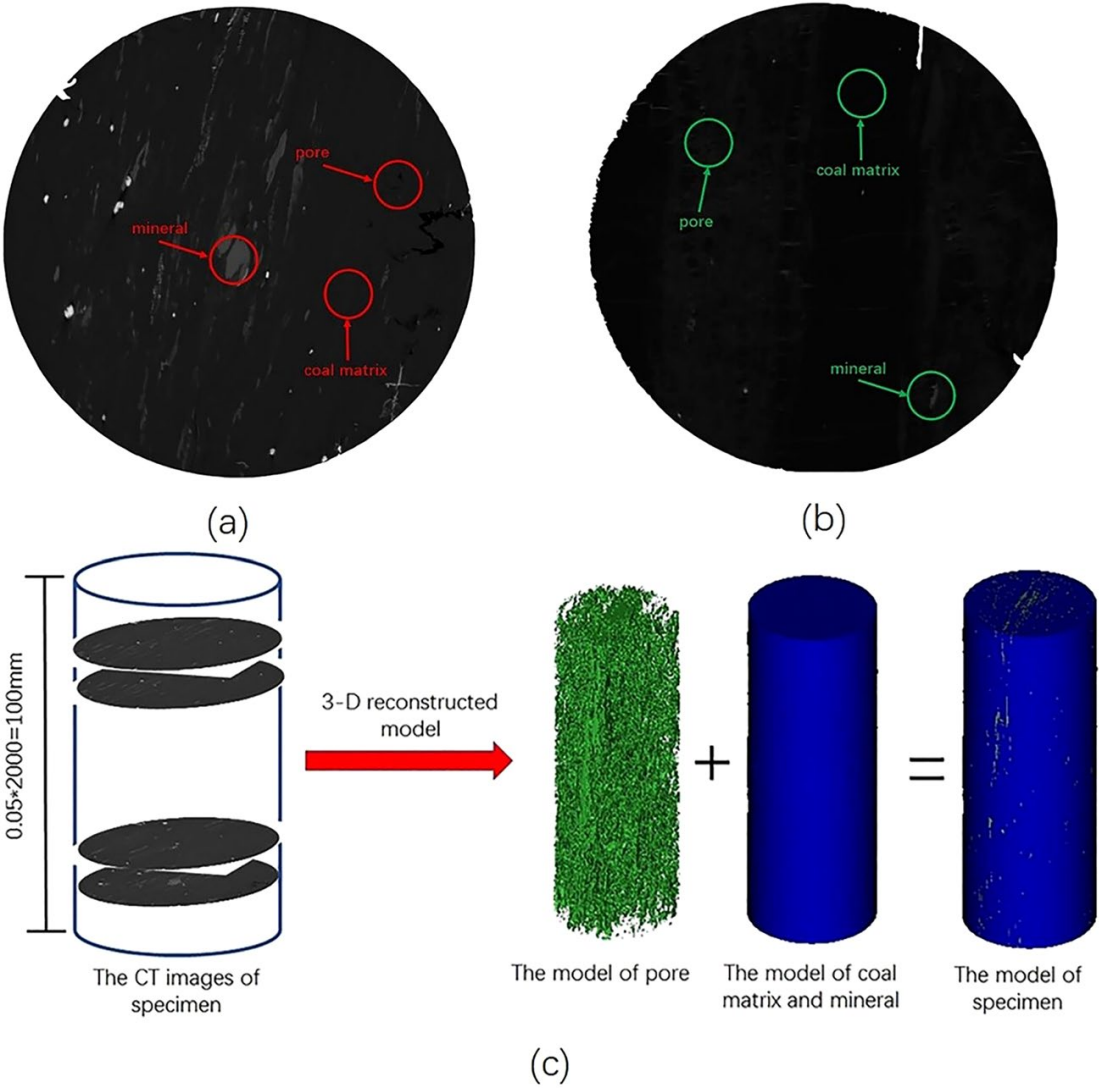
Images obtained by CT and the process of three-dimensional reconstruction. (**a**) CT schematic diagram of specimens from Hongqinghe Coal Mine; (**b**) CT schematic diagram of specimens from Nalinhe Coal Mine; (**c**) three-dimensional reconstruction sketch of CT image.

SEM provided high resolution two-dimensional images to describe the pore characteristics of the coal, but the specimens were small, and this method is destructive. Micro-CT compensated for these and images could be reconstructed for further analysis^38^. They were used to study pore connectivity. Part of the images obtained by SEM were provided in Fig. 11. Part of the images received by Micro-CT and the process of three-dimensional reconstruction were exhibited in Fig. 12. It could find out that most of the pore in the specimens from Hongqinghe Coal Mine were stomata, which was caused by gas accumulation and gas migration. Most of the pore in the specimens from Nalinhe Coal Mine were Tectonic pore, which was caused by geological structures and external loads. The pore interconnectivity is calculated by Mimics, the values of specimens from Hongqinghe Coal Mine and Nalinhe Coal Mine are 48.891% and 57.973%, respectively. Combined with results of mercury intrusion test and LTNAD test, it would draw the conclusion that the pore connectivity of specimens from Nalinhe Coal Mine were better.

## Conclusion

1. Coal with different bursting proneness show great difference in pore size distribution, pore connectivity, energy accumulation and dissipation during the uniaxial compressive deformation and damage. The failure of uniaxial compression increases the complexity of pore size distribution. The energy accumulation capacity of specimens with high bursting proneness is stronger than that of medium bursting proneness, the implication is more elastic energy at same strain.
2. Most of the pore in specimens with high bursting proneness is open pore, and the quantity of transition pore(10–100 nm), mesopore (100–1000 nm) and macropore (1000–10,000 nm) are great. The energy is inclined to accumulates under stress. The dominant pore in specimens with medium bursting proneness is transition pore(10–100 nm) and mesopore (100–1000 nm). The results show that the pore connectivity of specimens with medium bursting proneness are better.
3. Before and after uniaxial compression failure, the values of pore compressibility coefficient of specimens with high bursting proneness are larger under low pressure. However, they decrease significantly when the pressure increased. The values of pore fractal dimension of specimens with high bursting proneness are smaller. It indicates that the pore is more easily to deform.

## Data Availability

The data appeared in the manuscript were from experiment.
